# Impact of the COVID-19 Outbreak on the Antibiotic Use Patterns among a Rural Community Population in Eastern China

**DOI:** 10.3390/antibiotics11111544

**Published:** 2022-11-03

**Authors:** Bingbing Zhu, Xinping Zhao, Yurong Li, Na Wang, Helen Lambert, Fei Yan, Qingwu Jiang, Chaowei Fu

**Affiliations:** 1 Key Laboratory of Public Health Safety, NHC Key Laboratory of Health Technology Assessment, School of Public Health, Fudan University, Shanghai 200032, China; 2 Population Health Sciences, Bristol Medical School, University of Bristol, Bristol BS8 2PS, UK

**Keywords:** impact of the COVID-19 outbreak, antibiotic use, community population, rural China

## Abstract

There are growing concerns that the coronavirus disease of 2019 (COVID-19) pandemic may change antibiotic use patterns and accelerate antibiotic resistance, but evidence from the community level is lacking. This study aims to estimate the impact of the COVID-19 outbreak on the antibiotic use patterns among a community population in Eastern China. A self-administered medicine diary was used to collect information on antibiotic use from July 2019 to June 2021 among a rural community in Eastern China. We analyzed the changes in antibiotic use patterns over five months from August to December 2019 and the corresponding months in 2020. The risk of antibiotic use and its changes were measured with the incidence rate (IR) and relative risk (RR). In total, 1111 participants were eligible for the final analysis (440 in 2019 and 671 in 2020). After the COVID-19 outbreak, antibiotic use increased by 137% (5.43 per 100 person months in the 2019 vs. 12.89 per 100 person months in the 2020), and after the adjustment of covariates, the adjusted RR was 1.72 (95% CI: 1.10~2.34). It was higher among those who were women (RR = 2.62), aged 35–59 years old (RR = 2.72), non-farmers (RR = 2.75), had less than six years of education (RR = 2.61), had an annual household income over CNY 100,000 (USD 14,940) (RR = 2.60), and had no history of chronic diseases (RR = 2.61) (all *p* < 0.05). The proportion of cephalosporins consumed increased from 54.29% in 2019 to 64.92% in 2020 (*p* = 0.011). Among those aged 35 years and older, the proportion of antibiotics obtained from medical facilities increased, while the proportion obtained from retail pharmacies, homes, and other sources decreased (all *p* < 0.05). The COVID-19 outbreak changed antibiotic use patterns in this study population (Eastern China) significantly. More efforts to monitor and enhance antibiotic stewardship activities at the community level are needed in future.

## 1. Introduction

Both antimicrobial resistance (AMR) and COVID-19 are currently challenging global public health [1,2,3,4]. It was estimated that AMR would cause 10 million deaths per year by 2050 if no action were taken to stop its spread [5]. The current pandemic caused by COVID-19 poses an immense threat to global public health. Until 2nd August 2022, COVID-19 has infected almost 601 million people, resulting in over 6.5 million deaths globally [6]. Although it is a viral illness, patients with COVID-19 may develop severe complications associated with bacterial pathogens; hence, different antimicrobials, especially antibiotics, were used as empirical therapy [7,8,9,10,11]. Furthermore, during the early stages of the pandemic, there were insufficient effective antivirals with proven efficacy, which, combined with the anxiety and uncertainty of available treatments, led to the empirical but rather widespread and excessive use of antibiotics [11]. However, not all antimicrobial use in COVID-19 cases is warranted [12,13], and the increased use of antimicrobials is somewhat counterproductive in the global effort to reduce AMR [14]. A study in Italy showed that, during the lockdowns for COVID-19, there was a reduction in antibiotic consumption among children due to closed daycare centers and schools, while a relative increase in azithromycin use in adults was apparent [15]. However, there was no strong evidence for the routine use of azithromycin for reducing the time to recovery or risk of hospitalization from suspected COVID-19 in the community [16]. There is growing concern that due to the COVID-19 pandemic, the disruption of antimicrobial stewardship and infection prevention programs, inappropriate antibiotic treatment in patients with COVID-19, as well as the high number of patients and limitations of personal protective equipment, may increase the risk of AMR, although other actions, such as strict lockdowns, social distancing, and the extensive implementation of hand hygiene and face masks may decrease it [17,18,19,20,21,22].

Antibiotic use is one of the key drivers in the development of antibiotic resistance, with community and hospital settings being equal ecological niches of its emergence in human health [23,24]. At the user level, the “unjustified” or “irrational” behaviors of antibiotic use are thought to include failing to start a course of therapy, an omission of doses, the early cessation or unnecessary prolongation of therapy, errors in dose quantity, “inappropriate” intervals between doses, modifications to recommended regimes, and the use of shared prescriptions and medicines [25,26,27,28]. Self-medicating with antibiotics is framed as another “problematic” behavior in the development of antibiotic resistance [29,30,31]. Compared with the convenient location and timely access of pharmacies, the limited availability of physicians coupled with long waiting times in hospitals drive the self-purchasing of antibiotics, particularly in rural areas [32,33]. In this context, there has been easy access to antibiotics without a prescription, especially in rural China. The community’s perceptions of antibiotic use and AMR are very important for their control [34].

Moreover, emerging studies have highlighted the contribution of the COVID-19 pandemic to the escalation of AMR from multiple perspectives, such as extensive hospital antibiotic overuse and misuse [35,36,37,38] and the changes in total antibiotic consumption [39,40]. Controversially, some studies suggested that the use of several antibiotics increased in 2020 compared to 2019 [41,42,43,44,45], despite the fact that many studies reported that the total antibiotic consumption decreased during or after COVID-19 in Europe [40], the United States [46,47], Singapore [48], and China [39]. So far, few studies have focused on the impact of COVID-19 on antibiotic use among the community population [49,50]. Hogberg et al. reported that community antibiotic consumption decreased in nearly all EU/EEA countries during COVID-19 [49]. Furthemore, Kalam conducted a qualitative study to understand users’ perspectives on antibiotic seeking and consumption during COVID-19 [50]. Nevertheless, the changes in antibiotic use patterns were not clear, which may prompt evidence of how COVID-19 affects antibiotic resistance. Therefore, this study aimed to estimate the impact of the COVID-19 outbreak on the overall antibiotic use patterns among the rural community population in Eastern China as reported in self-administered medication diaries.

## 2. Results

### 2.1. Characteristics of Participants 

A total of 1111 eligible participants (440 in 2019 and 671 in 2020) had similar general characteristics between 2019 and 2020. More than half were female, with an average age of 59.17 ± 14.07 years. Most of them were non-farmers, had less than six years of education, and reported no history of chronic diseases. Moreover, more than half had an annual household income over CNY 100,000 (USD 14,940). Compared to 2019, the participants in 2020 had a significantly higher proportion of participants that were 60 years and older (55% vs. 48%), but a lower proportion of farmers (35% vs. 41%) and an annual household income over CNY 100,000 (43% vs. 63%) (Table 1).

### 2.2. Changes in the Risk of Antibiotic Use between 2019 and 2020

Overall, the incidence rate of antibiotic use was 5.43 per 100 PMs in 2019, prior to the COVID-19 outbreak in China, and 12.89 per 100 PMs in 2020 in the post-period, respectively. The risk of antibiotic use was 2.37 times greater in 2020 than in 2019 (*p* < 0.001). After the adjustment of covariates, including gender, age groups, education levels, farmer, annual household income, and chronic disease history, the adjusted RR was 1.72 (95% CI: 1.10~2.34) in the Cox regression model. There was a greater increase among those who were women, aged 35–59, farmers, had less than 6 years of education, had an annual household income over CNY 100,000 (USD 14,940), and had no history of chronic diseases. However, this increase was not found among the participants aged 15–34 years old (Table 2).

### 2.3. Changes in Antibiotic Categories and Access

**Figure 1:**
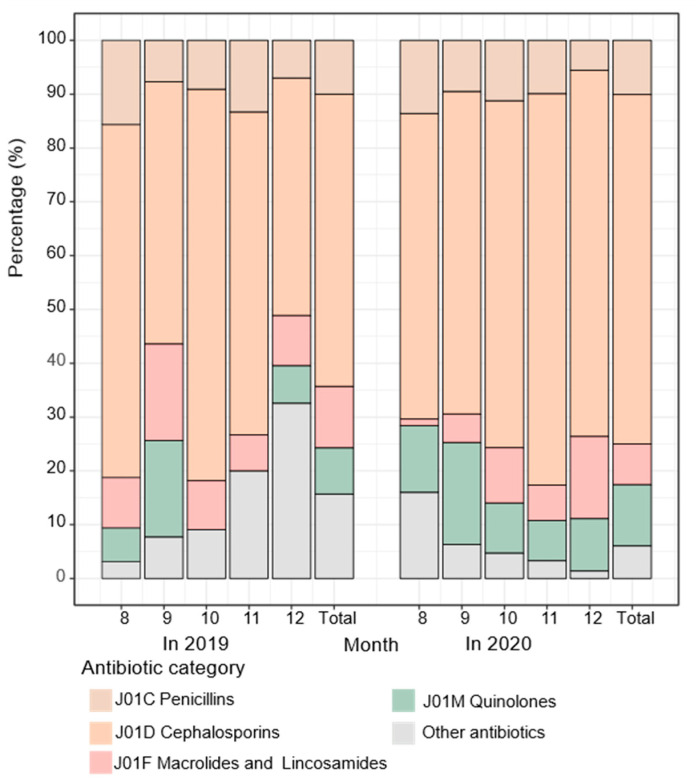
The proportion of different categories of antibiotics in medicine diaries among the study population in Eastern China, 2019–2020. (Data shown are unstandardized percentages. The proportion of cephalosporins are different between 2019 and 2020 (*p* = 0.011).).

**Figure 2:**
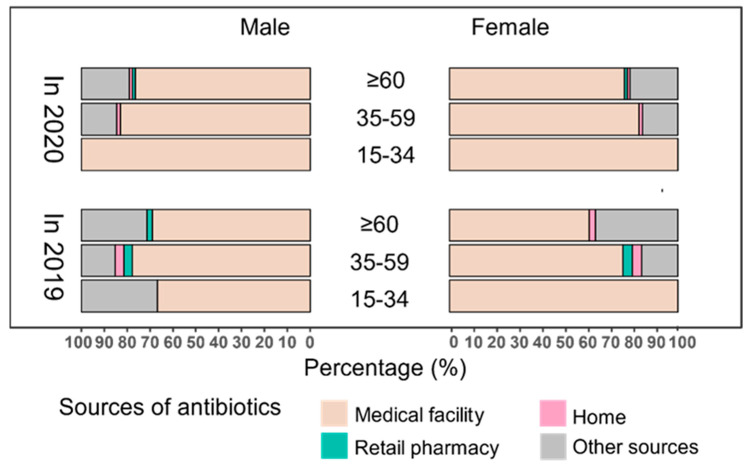
Sources of antibiotics in medicine diaries among the study population in Eastern China, 2019–2020. (Data shown are unstandardized percentages. In total, the sources of antibiotics changed significantly after COVID-19 (*p* = 0.002)).

**Figure 3:**
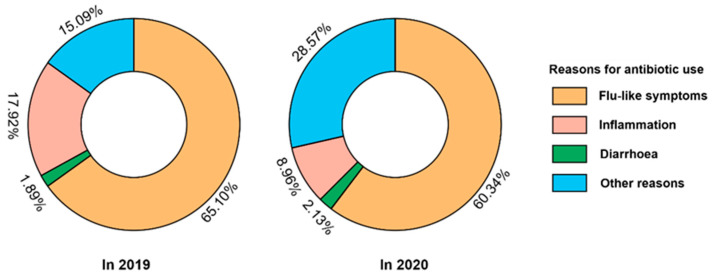
Reasons for antibiotic use in medicine diaries among the study population in Eastern China, 2019–2020. (Data shown are unstandardized percentages. In total, the sources of antibiotics changed significantly after COVID-19 (*p* = 0.005)).

Cephalosporins were always the most used antibiotics, and the proportion increased significantly from 54.29% in 2019 to 64.92% in 2020 (*p* = 0.011), but quinolones also increased, and macrolides and lincosamides decreased without significance (Figure 1). In 2019, the proportions of antibiotics obtained from medical facilities, retail pharmacies, homes, and other sources were 69.29%, 2.14%, 2.14%, and 26.43%, respectively, and 82.35%, 0.21%, 1.05%, and 16.39% in 2020, with medical facilities being the principal source of antibiotics. After the COVID-19 outbreak, the proportion of antibiotics obtained from medical facilities increased, while the proportion obtained from retail pharmacies, homes, and other sources decreased among those aged 35–59 and 60 years and older (all *p* < 0.05). Furthermore, the subgroup analysis revealed that among females aged 60 years and over, the proportion of antibiotics obtained from medical facilities increased (*p* = 0.013), while the proportion obtained from homes (*p* = 0.035) and other sources (*p* = 0.031) decreased, whereas males showed no significant changes in any age group (Figure 2). In addition, the reasons for antibiotic use reported by the subjects changed significantly after the COVID-19 outbreak (*p* = 0.005). Lower proportions were reported for treating flu-like symptoms (65.10% in 2019 vs. 60.34% in 2020) and inflammation (17.92% in 2019 vs. 8.96% in 2020), while higher proportions were reported for treating diarrhea (1.89% in 2019 vs. 2.13% in 2020) and other symptoms (15.09% in 2019 vs. 28.57% in 2020) after the COVID-19 outbreak. However, the top two reasons were treating flu-like symptoms and inflammation (Figure 3).

## 3. Discussion

### 3.1. Summary

The COVID-19 pandemic has severely impacted healthcare delivery, and there are growing concerns that the pandemic may change antibiotic use patterns and accelerate antibiotic resistance [23,24]. Thus, determining the impact of COVID-19 on antibiotic use patterns would help us to understand the impact of antibiotic resistance among community populations. In this community-level study among a rural population in Eastern China, the COVID-19 outbreak changed antibiotic use patterns significantly, including the risk for antibiotic use and the consumption proportion of cephalosporins. Additionally, the proportion of antibiotics obtained from medical facilities increased among certain groups of subjects, and the reasons for antibiotic use slightly changed.

### 3.2. The Potential Reasons Why the Risk of Antibiotics Increased after the COVID-19 Outbreak

Although the impact of the COVID-19 outbreak on antibiotic consumption has been documented in some epidemiological studies, few of them were conducted among community populations because the reliable data on antibiotic use are largely limited to hospitalized patients [39,40,49,50]. Our study estimated that the risk of antibiotic use increased by 137% after the COVID-19 outbreak in China, while it was reduced to 72% after the control of the covariates. This is particularly significant given that no confirmed COVID-19 cases were reported in the region during the study period. A study in Bangladesh showed an increased antibiotic use rate for illnesses reported in the preceding four weeks at the community level during the COVID-19 pandemic [51]. Another study in Italy showed that, during the lockdowns for COVID-19, there was a relevant reduction in antibiotic consumption among children due to closed daycare centers and schools, except for a relative increase in azithromycin use in adults [15]. This finding from our study can be explained. During the COVID-19 pandemic, the general population was likely sensitized regarding the use of antibiotics for treating COVID-19. According to the web-based surveillance of antibiotics used in Malaysia, 37% of the participants were aware that using antibiotics could not speed up recovery from all infections [52]. Still, 49% of the respondents reported that antibiotics were only effective against bacterial infections [52]. August to December 2020 (post-COVID-19 outbreak) was a relatively “peaceful” period, during which the government’s priority was vaccination for COVID-19. However, the perceived need for antibiotics may increase during the pandemic due to health anxiety. In such circumstances, doctors might agree to prescribe antibiotics, allowing people to obtain antibiotics easily. Another study elsewhere in Asia indicated that antibiotic-seeking behavior was related to a perceived vulnerability, management of an “unknown” illness, and anxiety [50], while an earlier Dutch study showed that if patients expected an antibiotic prescription, they were more likely to receive one [53]. Correspondingly, our study also showed that the proportion of medical facility-sourced antibiotics increased after the outbreak, in part, perhaps, because the availability of antibiotics purchased over the counter was increasingly restricted after the start of the pandemic. Additionally, there has been a surge in self-medication activities since the COVID-19 pandemic because of the societal perception of risk and the urge to accomplish something for preventative and curative reasons [54,55,56]. A recent systematic review revealed that the prevalence of COVID-19-related self-medication was 44.9%, and 79% of them were antibiotics [57]. This finding implies that the COVID-19 pandemic may increase the risk of antibiotic resistance.

### 3.3. A More Pronounced Impact Occurred in Some Groups

Moreover, it was observed that there was a greater increase in the risk of antibiotic use among those who were women, aged 35–59 years old, non-farmers, had less than 6 years of education, had an annual household income over CNY 100,000, and had no history of chronic diseases. The theory of planned behavior (TpB) suggests that a person’s actual use of antibiotics is best predicted by their intentions, which are influenced by attitudes, subjective norms, and perceived behavioral control [58]. Given that women are more likely to acquire gynecological infections, they require more antibiotics. A European study reported that women and those with lower levels of education were more likely to be prescribed antibiotics [59]. A previous study in rural China suggested that middle age was a potential factor related to higher levels of antibiotic utilization while younger people used antibiotics more strictly and appropriately, which concurs with our study findings [60]. In addition, those who were non-farmers or had a higher household income had a higher risk of using antibiotics, partly because their interactions between relatives and friends may foster a culture of antibiotic use and provide more chances of antibiotic exposure within this population. Subjects with chronic diseases may have good knowledge about antibiotics and use them cautiously [61,62]. These findings call for specific community-level interventions on antibiotic use for certain socio-demographic groups in rural China.

### 3.4. Categories and Access of Antibiotic Use Changed Moderately

It was found that the proportion of cephalosporins increased significantly in this study population in 2020 compared with 2019, which was supported by previous studies [44,50]. A recent study reported that the use of third-generation cephalosporins increased by 19% in 2020 compared with 2019 [38]. Another study found an increase in hospital consumption, but only the consumption of ceftaroline increased significantly [44]. The dispensing of antibiotics increased profoundly in Egypt during the early period of the COVID-19 pandemic without proper clinical evaluation, and azithromycin, ceftriaxone, and linezolid were the major antibiotics used [63].

Medical facilities were the main source of antibiotics in both study periods. Compared to 2019, the proportion of antibiotics accessed from medical facilities increased in 2020, while the proportion obtained from retail pharmacies, homes, and other sources decreased among those aged 35–59 and 60 years and over in this study. A previous study revealed that 57% of retail pharmacies sold antibiotics without a prescription in the Zhejiang province in 2017 [64]. However, in order to monitor the possible COVID-19 cases, retail pharmacies were not allowed to sell antibiotics in Deqing County [65], which would greatly limit the purchase of antibiotics from retail pharmacies, and in turn, this is likely to have promoted more attendance at medical facilities and the consequent purchases of prescribed antibiotics from their pharmacies. This suggests that a stricter enforcement of policies would have great significance for rationalizing antibiotic use, and it seems to be a great opportunity for improving antibiotic management after the outbreak of COVID-19.

### 3.5. Strengths and Limitations

To our limited knowledge, this was the first study to describe the impact of the COVID-19 outbreak on the antibiotic use patterns among the community population by analyzing the findings from self-administered medicine diaries. This study has several strengths. First, it was based on a community population and covered prior- and post-COVID-19 outbreak periods in China. Secondly, a self-administered medicine diary may more comprehensively reflect the individual and household antibiotic use in the context of sharing antibiotics with families or friends and more accurately document antibiotic access from non-medical facilities in rural China [60]. However, this study has several notable limitations. Firstly, there may be a reporting bias in the antibiotic records, especially in those unable to write, although health professionals from the village clinic helped participants document all their medication use weekly. Secondly, the participants were also asked to retain all medicine packets, including any antibiotics, and these were checked by health professionals on a monthly basis. However, the use of local health professionals to monitor the study may have urged some participants to be reluctant to disclose instances of self-medication with antibiotics that involved a procurement from sources other than the village clinic. Thirdly, due to the restriction of funds, the authorities, and the pandemic, the purpose of antibiotic use was not included, which may be important to interpret the impacts of the COVID-19 outbreak. A further limitation is that this study was carried out in Deqing County, one of the most developed areas in China, so the findings should be extended with caution to other populations in China, especially for the developing areas. Additionally, this study only covered five months of one year, so variations over the whole year were not captured. 

## 4. Materials and Methods

### 4.1. Study Design and Population

(1) Study design: This study was from research carried out in a rural community in Eastern China in June 2019, as presented in our previous paper [66]. Data collected from August to December 2019 was the “prior” period of the COVID-19 outbreak in China, and those during the same five months in 2020 were the “post” period, during which no local cases of COVID-19 infection were reported in Deqing County.
(2) Sampling method: Based on the population cohort previously established in Deqing County of the Zhejiang province in China, one village was selected as the study site using the rapid cluster sample survey methodology [67], and a total of 1112 residents were recruited into this study (441 in 2019 and 671 in 2020). One individual had missing data for most variables in 2019 and was therefore excluded. Finally, 1111 participants were eligible for the final analysis (440 in 2019 and 671 in 2020).
(3) Sample size estimation: According to a study in the United States, the antimicrobial use prevalence was 8.2% [68]; we conservatively expected that the prevalence was 9.0% among this study population. A two-sided test was used with the following parameters: set α = 0.05, δ = 2.0%. Then the values were inputted into the following formula:
  (1) $$\;n=Z_{\alpha /2}\times P\times \left(1-P\right)/\delta^{2}=400$$
  Considering the non-response variable and increasing the sample size by 10%, the minimum sample size was estimated to be 440.
(4) The inclusion criteria were as follows: (1) aged 15 years old and over, (2) lived in the study sites for no less than six months, and (3) consented to inclusion in the study.
(5) Ethical statement: All participants read a consent form (including the study objectives, risks, and benefits for the participants, the confidentiality, and that participation was voluntary) and received clarification if requested before giving their oral consent to participate in the study. This study was reviewed and approved by the Institutional Review Board of the School of Public Health, Fudan University, China (number IRB#2019-03-0733).

### 4.2. Collections for General Information and Antibiotic Use

Two surveys in June 2019 and July 2020 using the same questionnaire through a face-to-face interview were carried out to collect information on the demographic variables (sex, age, ethnicity, education level, marriage status, occupation, and annual household income) and the history of chronic diseases in the same site. The details of the questionnaire were described in our study protocol paper [66]. The education level was the highest grade completed by the participant and was described as ≤6 years (elementary school and below) and >6 years (junior school and above). The chronic disease history covered seven common chronic diseases (including diabetes mellitus, hypertension, coronary heart disease, stroke, chronic kidney disease, chronic obstructive pulmonary disease, and cancers). 

To describe the patterns of antibiotic use in the population, a structured self-administered diary was used to collect the medicine use (antibiotics and chronic disease medicine) and individual health status in each participating household over a 12-month period, as described in our study protocol paper [66]. The data on the medicines used for each household member were recorded weekly by participants using the structured form to capture the types, reasons, sources, frequencies, doses, side effects, and other details of both antibiotic use and the use of other medicines for long-term conditions. The options for the antibiotic sources included: inpatient/outpatient services in medical facilities (including village clinics, township healthcare centers, and hospitals) with prescriptions; medical stores or pharmacies; homes (including family members or saved from a previous time); and others (including friends, online, stalls, or hawkers, and somewhere or someone else). The purposes of antibiotic use included: flu-like symptoms (including cold, fever, and cough), inflammation (including urinary, digestive, respiratory, and motor system inflammation), diarrhoea, and other reasons (including pain, sore throat, vomiting, dizziness, etcetera).

Antibiotics were subsequently categorized using the anatomical therapeutic chemical (ATC) classification codes [69]. The study drew on the medicine diary for the antibiotic use data and from the surveys for the socio-demographic data on the participants.

### 4.3. Statistical Analysis

For the descriptive statistics, the variables were described as percentages for the categorical variables and means ± SD for the continuous variables. The categorical variables were compared over the groups using the Chi-square test, and the continuous variables were analysed using the Student’s *t*-test.

Given that the risk of antibiotic use varies with the length of time for observation, we applied the incidence rate (IR) to estimate it in this study, and the difference between the subgroups was estimated with the relative risk (RR). The IR and RR of the antibiotic use were defined as the following:

(2) $$Incidence\;Rate=\frac{Number\;of\;subjects\;who\;used\;antibiotic}{\sum Time\;spent\;in\;this\;population}$$

(3) $$Relative\;Risk=\frac{Incidence\;Rate_{2020}}{Incidence\;Rate_{2019}}$$

in which the person-months (PMs) of the follow-ups were calculated from the date initially recorded to the date of the follow-up or end of the follow-up, whichever came first. The Z test was occupied for the incidence rate difference [70]. After controlling for gender, age, farmer, annual household income, and chronic disease history, the adjusted RRs were estimated in the Cox regression model. All the statistical tests were two-sided, and a *p* < 0.05 was considered statistically significant. All analyses were performed in R software (Version 4.1.0; R Foundation for Statistical Computing, Vienna, Austria).

## 5. Conclusions

In conclusion, this study identified an increase in the risk of antibiotic use, although the proportion of antibiotics obtained from medical facilities (and, by implication, via a prescription) increased among this rural community after the COVID-19 outbreak. It suggests that there was a window for improving antibiotic management during the pandemic. To ensure the appropriate use of antibiotics and to control AMR, more efforts need to be forged to monitor and improve antibiotic stewardship activities at the community level. More education and awareness campaigns and activities may be worthwhile, especially for older and less well-educated rural Chinese women who are otherwise healthy, since this group were found to be more likely to use antibiotics. 

## Figures and Tables

**Table 1:** General characteristics of the participants among the study population in Eastern China by years.

Characteristics	Total	2019	2020	χ^2^ or t	*p* Value
	n = 1111	n = 440	n = 671		
Gender					
Male	533 (47.97)	217 (49.32)	316 (47.09)	0.441	0.506
Female	578 (52.03)	223 (50.68)	355 (52.91)		
Age (years; mean ± SD)	59.17 ± 14.07	57.63 ± 14.70	60.14 ± 13.57	35.3	0.004
15–34	68 (6.12)	34 (7.73)	34 (5.07)	6.630	<0.036
35–59	464 (41.76)	195 (44.32)	269 (40.09)		
≥60	579 (52.12)	211 (47.95)	368 (54.84)		
Education (years) *					
≤6	768 (69.13)	297 (67.50)	471 (70.19)	0.782	0.377
>6	343 (30.87)	143 (32.50)	200 (29.81)		
Farmer *					
No	687 (62.74)	249 (58.73)	438 (65.28)	4.491	0.034
Yes	408 (37.26)	175 (41.27)	233 (34.72)		
Annual household income (CNY) *					
≤100,000	491 (49.25)	144 (36.64)	347 (57.45)	40.419	<0.001
>100,000	506 (50.75)	249 (63.36)	257 (42.55)		
History of chronic diseases					
No	615 (57.32)	243 (60.45)	372 (55.44)	2.377	<0.123
Yes	458 (42.68)	159 (39.55)	299 (44.56)		

Note: * Missed data; Abbreviations: CNY, Chinese Yuan.

**Table 2:** Changes in the risk of antibiotic use between 2019 and 2020 among the study population in Eastern China.

Characteristics	2019			2020			RR	Z Value	*p* Value
	Person Months	Cases	IR (100 PMs)	Person Months	Cases	IR (100 PMs)			
Overall	2116	115	5.43	3273	422	12.89	2.37	77.981	<0.001
Gender									
Male	1020	59	5.86	1520	187	12.30	2.10	33.276	<0.001
Female	1095	56	5.11	1753	235	13.41	2.62	43.558	<0.001
Age (years)									
15–34	147	4	2.72	152	7	4.61	1.53	1.082	0.280
35–59	914	41	4.49	1205	147	12.20	2.72	32.079	<0.001
≥60	968	66	6.82	1754	252	14.37	2.11	39.053	<0.001
Education (years) *									
≤6	1384	71	5.13	2203	295	13.39	2.61	54.904	<0.001
>6	645	40	6.20	908	111	12.22	1.97	19.937	<0.001
Farmer *									
No	1130	53	4.69	1993	257	12.90	2.75	44.457	<0.001
Yes	899	58	6.45	1117	149	13.34	2.07	30.338	<0.001
Annual household income (CNY) *									
≤100,000	624	42	6.73	1618	230	14.22	2.11	26.518	<0.001
>100,000	1185	53	4.47	1195	139	11.63	2.60	36.663	<0.001
History of chronic diseases									
No	1148	57	4.97	1695	220	12.98	2.61	43.43	<0.001
Yes	724	39	5.39	1416	186	13.14	2.44	28.76	<0.001

Note: * Missed data; Abbreviations: CNY, Chinese Yuan.

## Data Availability

The data presented in this study can be made available upon request from the corresponding author. The dataset is not publicly available as it forms part of ongoing research and analysis. It will be made publicly available once the major study publications are completed.
